# Growing Heart Valve Implants for Children

**DOI:** 10.3390/jcdd10040148

**Published:** 2023-03-31

**Authors:** Haley Konsek, Curry Sherard, Cora Bisbee, Lillian Kang, Joseph W. Turek, Taufiek K. Rajab

**Affiliations:** 1 Department of Surgery, College of Medicine, Medical University of South Carolina, Charleston, SC 29425, USA; 2 Department of Surgery, Duke University Medical Center, Durham, NC 27710, USA; 3 Division of Cardiovascular and Thoracic Surgery, Department of Surgery, Duke University Medical Center, Durham, NC 27710, USA; 4 Section of Pediatric Cardiothoracic Surgery, Department of Surgery, Medical University of South Carolina, Charleston, SC 29425, USA

**Keywords:** congenital heart disease, valve replacement, tissue-engineered heart valve, partial heart transplant

## Abstract

The current standard of care for pediatric patients with unrepairable congenital valvular disease is a heart valve implant. However, current heart valve implants are unable to accommodate the somatic growth of the recipient, preventing long-term clinical success in these patients. Therefore, there is an urgent need for a growing heart valve implant for children. This article reviews recent studies investigating tissue-engineered heart valves and partial heart transplantation as potential growing heart valve implants in large animal and clinical translational research. In vitro and in situ designs of tissue engineered heart valves are discussed, as well as the barriers to clinical translation.

## 1. Introduction

Congenital heart disease (CHD) is one of the most commonly diagnosed congenital diseases in newborns [1]. In the United States, 8–10 per 1000 newborns are affected with CHD [2]. Despite the increasing number of people living with CHD, 180,000 neonates and infants die each year from congenital heart disease [3]. Most of these deaths are caused by congenital valvular disease, which comprises 25% of all CHD diagnoses [4,5]. Surgical intervention is often indicated within the first year of life for the survival of patients with congenital valvular disease [6]. The current standard of care for infants and neonates with unrepairable valvular disease is heart valve replacement [4]. This can be accomplished using various techniques, including mechanical valves, bioprosthetic valves, cryopreserved homografts, and decellularized allografts. However, each of these approaches has significant drawbacks, especially in pediatric patients [7]. Mechanical valves are thrombogenic and therefore require lifelong anticoagulation, putting patients at an increased risk of hemorrhage and thromboembolic events [8,9]. Bioprosthetic valves are prone to structural valve degeneration. This is especially dangerous for the pediatric population, who are at higher risk for early structural valve degeneration and, consequently, earlier reoperation to replace the damaged valve [10]. Cryopreserved homografts become immunogenic in most patients with the creation of anti-HLA antibodies. Studies have shown that this immune reaction is stronger in infants and children with a decreased time to reintervention than in adults [11].

The most significant drawback to current valve replacement options is the inability of the implant to accommodate the somatic growth of the recipient. The currently available replacement strategies have a fixed functional diameter and ultimately result in acquired patient-prosthesis mismatch [12]. This requires pediatric patients to undergo multiple invasive re-operations to exchange the smaller valve for a larger one. Patients under 2 years old who undergo heart valve replacement commonly undergo up to 5 open heart surgeries during their lifetime, which has been linked to a greater risk of mortality [13,14].

Therefore, there is an urgent need for a heart valve implant that grows with the recipient, along with other essential characteristics, including non-thrombogenic, nonobstructive, and able to undergo remodeling to repair injury and maintain function [13,15]. Most of the research attempting to combat this issue has revolved around tissue engineering; however, these valves have failed to succeed in clinical translation [13]. Therefore, the aim of this review is to provide an overview of the published evidence on heart valve replacement efforts that accommodates the somatic growth of the recipient. This review will provide a summarization of cell sources and scaffolds used in tissue engineering, as well as the preclinical and clinical trials for in vitro and in situ tissue-engineered heart valves with growth potential (Table 1). The review will conclude with a summarization of partial heart transplantation as a possible alternative solution (Table 1). The specific search strategy is outlined in the Supplementary Materials.

## 2. Current Standard of Care

All currently available heart valve implants have limitations preventing long-term clinical success in pediatric patients. Mechanical heart valves are durable in vivo, but their thrombogenicity requires lifelong anticoagulation [8]. The risk of severe bleeding or thromboembolic event is 1% per patient per year [13]. The outgrowth of the mechanical valve causes deterioration in ventricular function and requires risky reoperations to replace the outgrown valve [8]. For patients with contraindications to anticoagulation, the American College of Cardiology and the American Heart Association recommend bioprosthetic heart valve implants [37]. These porcine valves are pretreated with glutaraldehyde to decrease immunogenicity, which decreases their durability in vivo, and they are thus prone to structural valve regeneration. This irreversible process leads to valve failure and requires a redo operation [10]. Patients less than 20 years of age are six times more likely to undergo valve failure, and the risk of reintervention is five times greater than in adults [38].

The current heart valve implants available for neonates are exclusively homografts because mechanical and bioprosthetic valves are not small enough for the patient population. To make the homografts widely available for use, frozen cryopreserved homografts are banked and stored below −135 °C in vapor-phase nitrogen [39]. Small amounts of viable cells are retained in these valves after implantation that are immunogenic and incapable of biological functions such as growth and self-repair [40,41,42,43,44]. Consequently, cryopreserved homografts fail rapidly and require replacement as early as a few months after the initial operation. The mortality rate of aortic valve homografts in infants and neonates is 40%, and the mortality rate in primary truncus valve replacement is 75% [45,46].

Orthotopic heart transplants provide another treatment option for children with valvular disease. Although immune suppression is required, heart transplants accommodate the somatic growth of the recipient [47,48]. The valves of the transplanted heart keep normal cellularity and architecture for biological functions and self-repair, and short-term outcomes of neonatal heart transplants have revealed less than 5% mortality [49,50,51]. Despite these benefits, chronic myocardial changes of orthotopic heart transplants lead to graft failure over time, with a mortality rate of 35–50% by 20 years [51,52].

## 3. Strategies for Delivering Growing Heart Valves

### 3.1. Tissue-Engineered Heart Valves

The treatment of heart valve dysfunction in neonates and infants remains an unsolved problem. There is an urgent clinical need for growing heart valves for pediatric patients; however, all attempts at creating a growing heart valve implant have failed in clinical translation. Tissue engineering has been at the forefront of research for creating the ideal heart valve replacement. The basic concept is to create a functionally viable tissue that resembles the native valve and is capable of growth, remodeling, and repair. This requires a 3D scaffold, cells to seed the scaffold, and appropriate biomolecules or bioreactors to allow the proliferation of these cells onto the scaffold [53]. This has been studied using various combinations of cells, scaffolds, and seeding methods, including in vitro, in situ, and in vivo.

#### 3.1.1. Design—Cell Sources and Scaffolds

Cells populating heart valves play an important role in maintaining long-term durability by remodeling the extracellular matrix to repair damage caused by the repetitive stress and movement of the leaflets. Cell sources for TEHV can be xenogenic, allogenic, or autologous. The optimal cell source for clinically translational TEHV is autologous rather than the immunogenic xenograft and allograft [54]. Early studies on autologous cell populations for tissue engineering determined that patient-derived endothelial and interstitial cells are crucial for valve integrity and tissue homeostasis [55], leading to many preclinical studies utilizing these cell types in TEHV design.

Further research has been conducted testing autologous stem cells as a viable alternative cell population for tissue engineering. Non-hematopoietic bone marrow-derived cells, mesenchymal cells, amniotic fluid cells, umbilical cord cells, and induced pluripotent stem cells (iPSCs) have been considered for study [54,55]. However, TEHVs utilizing these cell sources have shown little promise in preclinical studies [54]. For example, Gottlieb et al. created an in vitro-engineered pulmonary valve seeded with ovine bone marrow-derived mesenchymal stem cells [16]. The valves were successfully implanted into the donor sheep; however, all valves developed severe regurgitation after 12–20 weeks. Furthermore, valve growth was not observed in the sheep models, but the authors were unclear on whether this represented an actual lack of growth versus a short follow-up time [16].

Another important consideration when designing TEHVs is the scaffold. Scaffolds provide a 3D support platform for cell adhesion, growth, and tissue formation [56]. They can be classified into two main categories: resorbable biomaterials and decellularized xenografts/allografts. Bioresorbable polymeric scaffolds are made from natural biomaterials, synthetic biomaterials, or a combination. Natural biomaterials, including collagen, fibrin, and gelatin, are non-toxic, fast degrading, and non-immunogenic [57]. Synthetic biomaterials include hydrogel polymers such as poly(ethylene glycol) (PEG) and poly(vinyl alcohol) (PVA), as well as hydrolytically degradable polymers such as poly(glycolic acid) (PGA) [54]. Bioresorbable scaffolds have drawn attention due to their ability to allow the design of valves with optimal characteristics, such as size and shape, as well as their biodegradable and mechanical properties [56].

Decellularized scaffolds made from decellularized xenogenic or allogenic valve tissue are repopulated by autologous cells in vivo. These scaffolds provide maintained tissue morphology, mechanics, and leaflet size as well as non-immunogenicity [56]. However, the tissue must be fully decellularized to prevent an immune response without damaging the structure to maintain function. Furthermore, repopulation with autologous cells in vivo has not proven definitive growth potential because the autologous cells do not behave like native valve cells [54].

#### 3.1.2. In Vitro Heart Valve Tissue Engineering

**Figure 1:**
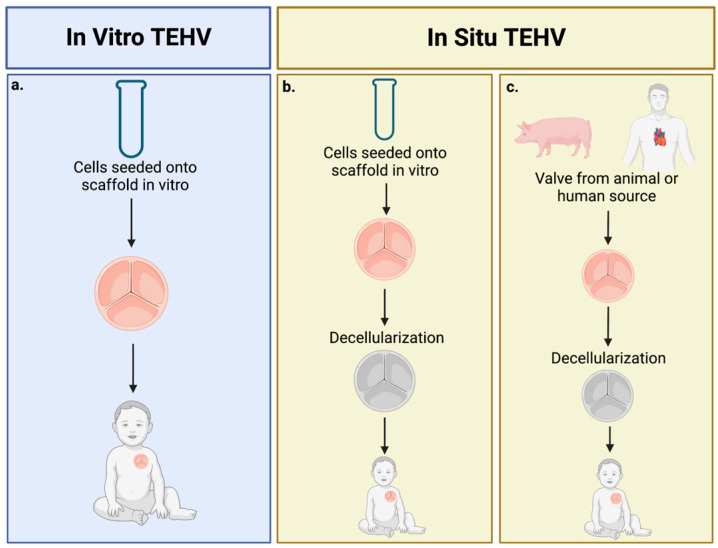
Overview of design methods for tissue engineering heart valves. (**a**) In vitro TEHV involves seeding cells on the scaffold in vitro, then delivering them to the patient. (**b**) In situ engineering using a decellularized in vitro designed valve. (**c**) In situ TEHV using a decellularized xenograft or allograft. Created with BioRender.com (accessed on 26 March 2023).

Many studies have attempted to create heart valves via in vitro heart tissue engineering. This method involves harvesting autologous cells from the patient, seeding 3D scaffolds in vitro, allowing tissue growth in vitro, and the implantation of the tissue into the patient (Figure 1a). The use of autologous cells suggests a promising strategy for valve growth; however, all in vitro methods to date have resulted in valve regurgitation and leaflet thickening when studied in vivo [16,17,18,58].

For example, large animal studies were conducted using harvested autologous vascular cells seeded onto a bio-polyester scaffold. After 17 weeks in vivo, the valves exhibited normal native valve function. However, the valves showed incomplete endothelial cell seeding, and there was inconclusive evidence of the overall cell growth of the valve [58]. Another investigation using an autologous fibrin scaffold seeded with autologous ovine endothelial, smooth muscle, and fibroblast cells demonstrated successful remodeling in vivo, however; all valves in this study eventually failed due to valvular insufficiency [17]. Additionally, the cell harvesting process for vascular-derived cell types is invasive and requires the resection of healthy vascular tissue. This procedure is contraindicated in patients with vascular disease and has limited application in growing pediatric patients [19]. Other barriers to clinical translation of the in vitro TEHV method include the high cost and regulations regarding cell isolation, expansion, and manufacture of TEHVs [59]. Schmidt et al. attempted to address some of these barriers by creating a completely minimally invasive approach [18]. Stem cells were isolated noninvasively and then seeded in vitro onto a scaffold within a self-expanding stent. The valves were successfully implanted into sheep using a minimally invasive transapical approach. After 8 weeks in vivo, the valves demonstrated normal functionality; however, leaflet thickening was also observed [18]. Therefore, more research must be completed before in vitro TEHV is clinically translatable for pediatric patients.

#### 3.1.3. In Situ Heart Valve Tissue Engineering

Another heavily studied method of cell seeding is the concept of in situ engineering (Figure 1b,c). This method includes the development of heart valve replacements that are acellularized and thus require endogenous cellularization by host cells in vivo. The major scaffolds with growth potential for in situ engineering are in-vitro TEHV which are decellularized before implantation, and acellularized polymeric scaffolds. These acellular models provide a less-expensive, off-the-shelf product with fewer regulatory barriers than cellular in vitro models [60].

The use of decellularized, in vitro scaffolds for in situ TEHV (Figure 1b) provides a nonimmunogenic scaffold in contrast to decellularized xenografts and allografts (Figure 1c) where the possibility of an immune reaction remains [60]. Weber et al. used this method to create a human fibroblast-derived decellularized TEHV implanted in non-human primates (chacma baboons) [20]. The valves showed rapid homogenous cellular repopulation by 4 weeks. At 8 weeks of follow-up, insufficient coaptation with radial leaflet shortening and moderate regurgitation was observed [20]. Transcatheter implantation approaches have also been tested using various decellularized TEHV designs implanted in sheep [21,22]. Driessen-Mol et al. tested a decellularized TEHV engineered on a synthetic scaffold with autologous vascular-derived cells [21]. These valves developed moderate regurgitation by 24 weeks post-op with a significant decrease in coaptation over time [21]. Motta et al. designed a decellularized TEHV with integrated Valsalva sinuses engineered with human neonate dermal fibroblasts [22]. These valves demonstrated optimal functionality after 4 h in vivo in a sheep model; however, follow-up time was not long enough to determine growth capacity [22]. To address the issue of growth, researchers created a decellularized tubular valve engineered in vitro, which has been implanted in both the pulmonary and aortic positions in sheep [23,24]. In the pulmonic position, normal valve integration and function were observed for the first 8 weeks after implantation. Between 11.9 and 21.9 weeks, every valve progressively lost its functional ability, underwent leaflet shortening, and became nonfunctional [23]. In the aortic position, implanted valves demonstrated extensive recellularization, stable valve performance, and no stenosis after 24 weeks [24]. Overall, the short follow-up times of these studies have yet to prove the long-term efficacy of in situ TEHV. The longest follow-up study to date was testing the long-term performance of a decellularized TEHV designed using a computational model for 1 year post-implantation in sheep. After 1 year, the valves demonstrated evidence of remodeling, preserved functionality, and mild regurgitation [25]. Despite these promising results, decellularized TEHVs engineered in vitro for in situ seeding are expensive and time-consuming, preventing their translation into clinical use [60].

The second in situ approach involves the use of bioresorbable polymeric scaffolds. These provide more affordable and readily available options for clinical use [60]. Pre-clinical studies of a novel tri-leaflet polymeric transcatheter pulmonary valve with balloon-expandable stent were performed in sheep models. After 4 weeks in vivo, these valves demonstrated normal function and shape. However, mild fibrous overgrowth was noted on the valve membrane and bottom of the leaflets, which is concerning regarding the long-term durability of the valve [61]. In 2016, a pulmonary heart valve scaffold created from a bioresorbable elastomer based on bis-urea-modified polycarbonate was implanted in sheep and followed for 2, 6, and 12 months. After 12 months, the valve implants demonstrated sustained functionality with evidence of host cell colonization and endogenous tissue formation. However, scaffold resorption was not complete after 12 months, indicating the need for a follow-up study to determine the long-term functionality of the valve. In addition, the valves showed leaflet thickening and neovascularization, which could indicate a potential risk of retraction and failure of the valves with time [26].

#### 3.1.4. TEHVs in Clinical Studies with Pediatric Patients

To date, the majority of TEHV tested in pediatric patients have consisted of in situ TEHV designs. A successfully growing TEHV was implanted in two pediatric human patients (ages 11 and 13 years) in 2002. The valves consisted of a decellularized human pulmonary valve allograft reseeded with autologous endothelial progenitor cells. After surgical implantation, the implanted valves demonstrated mild pulmonary regurgitation. However, 3.5 years later, echocardiography of the TEHVs revealed decreased regurgitation and no evidence of valve degeneration. Additionally, the valve annulus diameter increased, indicating somatic growth along with the children [27].

Decellularized xenografts implanted in the pulmonary position have failed rapidly in pediatric patients [28,29,30]. In contrast, decellularized allografts used for pulmonary valve replacement have shown promising results after 10 years, including reduced degeneration and decreased need for explantation when compared to the current standard of care. However, some of these implants developed stenosis and regurgitation. Additionally, the implants showed evidence of growth at mid-term follow-up, but further testing is required to prove adaptive growth and adequate recellularization [31].

There is limited clinical data involving pediatric patients and the implantation of decellularized allografts in the aortic position. The first clinical study involving pediatric patients under 10 years old was published in 2016 by Tudorache et al [32]. An average follow-up time of 2–3 years for these 16 patients revealed no observable increase in valve annulus diameter. An infant in this study developed subvalvular stenosis secondary to left ventricular outflow tract obstruction, which required reoperation. Tudorache et al. noted successful recovery of the infant as well as continued physiological development after 4.5 years [32]. Early results from a follow-up study called the ARISE trial included 28% of pediatric patients in the study population. The decellularized aortic allografts demonstrated comparable results to the Ross procedure after 2 years of follow-up, with pending results after the intended 10-year follow-up period [33].

A clinical trial completed in 2021 provided the first human trial of a bioresorbable pulmonary valve conduit in pediatric patients. The Xeltis pulmonary valve conduit is an electrospun polymeric heart valve composed mainly of bioresorbable supramolecular 2-ureido-4[1H]-pyrimidone. Twelve pediatric patients underwent transplantation and were followed for up to 24 months. The results of this study demonstrated no evidence of stenosis or valve degeneration with clinical improvement in all patients. However, these patients developed moderate to severe pulmonary regurgitation over time. Additionally, follow-up was not long enough to prove somatic growth of the valve with the recipient. Researchers are now testing a new design to address the challenge of regurgitation [34], but to date, this valve has not passed early clinical trials.

### 3.2. Partial Heart Transplant

**Figure 2:**
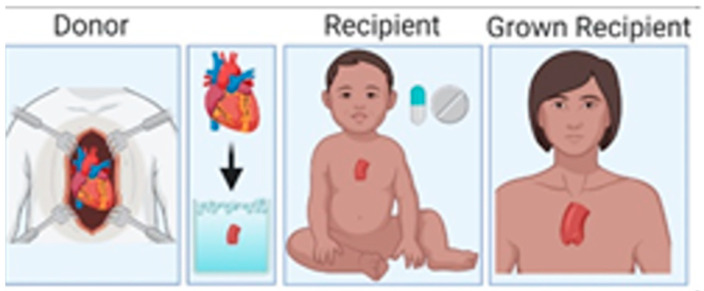
Partial heart transplantation involves transplanting the part of the heart containing the valve. Created with BioRender.com (accessed 1 November 2022).

A new approach to provide a solution for growing pediatric heart valve replacements is partial heart transplantation (PHT). This approach involves transplanting only the root of the heart valve from a donor heart (Figure 2). The transplant is tissue matched with the recipient, who is required to undergo immunosuppression until they have grown sufficiently to receive an adult-sized prosthetic valve. At this point, the patient would discontinue immunosuppression and begin anticoagulation. Therefore, successful partial heart transplants are expected to offer a normal life expectancy. This differs from conventional heart transplants, which invariably fail over time from allograft vasculopathy of the ventricles. Additionally, PHT contains live cells that accommodate the somatic growth of the patient, are capable of self-repair, and are non-thrombogenic [35]. 

Initial studies published on cold ischemia time of heart valve transplants revealed cellular viability within 48 h of cold storage versus conventional heart transplant with limited cold storage time of up to 6 h. This offers an increased procurement radius for partial heart transplants [62]. Additionally, transplantation of only the heart valve allows for procurement from diseased hearts with healthy valves that are not candidates for orthotopic transplantation. Thus, PHT would increase the number of heart donor candidates and help alleviate the current shortage of donor hearts for conventional heart transplants [63].

Researchers have published a clinical trial protocol for a prospective single-arm pilot trial to determine the feasibility and safety of PHT as well as analysis of valve annulus growth and evidence of valve regurgitation or stenosis. This trial will be performed on infants and children less than 2 years old in need of semilunar heart valve transplant [36].

## 4. Conclusions

In conclusion, a major gap in the treatment of pediatric patients with congenital valvular disease is a heart valve replacement that undergoes somatic growth with the recipient, is non-thrombogenic, nonimmunogenic, and maintains normal valvular function. Tissue-engineered heart valves have been studied extensively in order to provide a solution to this need; however, no tissue-engineered heart valves have succeeded in clinical translation. In vitro tissue-engineered valves have deteriorated in preclinical studies due to valve insufficiency [16,17,18,19]. Additionally, these valves are time-intensive and expensive, and the use of stem cells brings ethical barriers to clinical translation [55]. In situ tissue engineered heart valves provide an off-the-shelf supply that is less expensive than in vitro [60]. However, these valves have demonstrated limitations in preclinical and clinical trials, including inadequate cell adaptation and regurgitation [20,21,22,23,24,25,26,27,28,29,30,31,32,33,34,35,61]. Furthermore, the limited supply of correctly sized allografts presents a challenge for clinical use [34]. Partial heart transplantation offers an alternative replacement approach for neonates and infants with irreparable congenital valvular disease. 

## Figures and Tables

**Table 1:** Overview of recent preclinical and clinical evaluation of growing heart valves.

Stage of Development	Approach	Progress	Reference
In vivo	Autologous ovine bone marrow-derived mesenchymal cells seeded onto a bioresorbable scaffold	Acceptable initial valve function in sheep with increasing regurgitation and decreasing cusp length after 12–20 weeks	[16]
In vivo	Autologous vascular cells seeded on a biopolyester scaffold in vitro	Large animal studies in sheep revealed normal function after 17 weeks with mild stenosis and incomplete endothelial cell seeding	[17]
In vivo	Autologous endothelial, smooth muscle, and fibroblast cells seeded on patient-derived fibrin scaffold in vitro	Sheep studies revealed successful remodeling after 3 months; however, all valved failed due to valvular insufficiency	[18]
In vivo	Autologous ovine bone-marrow-derived stem cells seeded onto a bioresorbable scaffold integrated into a self-expanding stent	Minimally invasive implantation in sheep was successful. After 8 weeks, valves showed normal functionality with leaflet thickening present	[19]
In vivo	Decellularized heart valve fabricated on a bioresorbable nitinol stent scaffold with human vascular-derived fibroblasts	Prior to implantation, valves demonstrated reduced coaptation leading to leaflet shortening after implantation in non-human primates (chacma baboons)	[20]
In vivo	Decellularized heart valve engineered on a rapidly degrading synthetic scaffold with autologous vascular-derived cells	By 24 weeks post-implantation, moderate regurgitation was observed in sheep models with a significant reduction in coaptation leading to non-physiological loading and insufficient washout during diastole	[21]
In vivo	Decellularized valve engineered in vitro from human neonatal dermal fibroblasts on a bioresorbable PGA scaffold with integrated Valsalva sinuses	4 h after implantation in sheep, valves demonstrated normal function	[22]
In vivo	Decellularized tubular valve engineered in vitro from autologous ovine dermal fibroblasts with degradable sutures	Valve integration and normal function of implanted valves in sheep for 8 weeks with leaflet shortening, loss of functional ability and ultimately valve failure by 22 weeks	[23]
In vivo	Decellularized tubular valve engineered on a collagen scaffold with ovine dermal fibroblasts	24 weeks after implantation in the aortic position in sheep, valves showed normal function and recellularization	[24]
In vivo	Computationally inspired in vitro design of decellularized TEHV seeded with myofibroblasts	After 1 year of implantation in sheep, valves showed normal function, ECM remodeling, and mild regurgitation	[25]
In vivo	Trileaflet polymeric pulmonary valve with leaflets made of 0.1 mm expanded polytetrafluoroethylene coated with phosphorylcholine and balloon-expandable stent	Polymeric valves implanted in sheep exhibited normal function, and no evidence of insufficiency or thrombosis; however, mild fibrous overgrowth was revealed with no evidence of tissue infiltration	[26]
In vivo	Pulmonary valve with scaffold created from a bioresorbable novel supramolecular elastomer based on bis-urea-modified polycarbonate	Twelve months after implantation, valves demonstrated normal functionality with evidence of host cell colonization and formation of neo-tissue. However, scaffold resorption was incomplete, indicating longer follow-up studies for long-term durability	[27]
Clinical	Decellularized human pulmonary valve allograft reseeded with autologous endothelial progenitor cells	The valves were implanted in two pediatric patients. At 3.5 years follow-up, the valves demonstrated trivial regurgitation, increased valve annulus diameter, and no evidence of valve degeneration	[28]
Clinical	Synergraft^TM^ valve: Decellularized porcine heart valve	Hyperacute and acute rejection of valves, resulting in the deaths of 3 of the 4 children	[29]
Clinical	Decellularized xenograft using Matrix P plus (decellularized porcine pulmonary valve)	Six of the 16 pediatric patients required reoperation after 10 months due to graft obstruction secondary to inflammatory infiltration	[30]
Clinical	Decellularized xenograft using Matrix P and Matrix P plus pulmonary valves	Reoperation was required in 14 of 26 patients due to graft failure secondary to inflammation and fibrosis	[31]
Clinical	Decellularized pulmonary valve homograft	Ten year follow-up in pediatric patients revealed less degeneration than the current standard of care, but some implants developed stenosis and regurgitation; evidence of growth was present after 5 years	[32]
Clinical	Decellularized aortic allograft	Average 2–3 year follow-up in 16 pediatric patients revealed normal valve function but no evidence of annulus diameter growth	[33]
Clinical	ARISE trial: Decellularized aortic allograft	Early results in pediatric patients demonstrated comparable results to the Ross procedure, pending 10 year follow-up results	[34]
Clinical	Xeltis pulmonary valve made of bioresorbable supramolecular 2-ureido-4[1H]-pyrimidone	The Xeltis valve was transplanted into 12 human pediatric patients. After 24 months, the valves showed no evidence of degeneration or stenosis. However, 5 patients developed severe insufficiency due to leaflet prolapse	[35]
Clinical	Partial heart transplantation	Prospective, non-randomized, single-center, single-arm pilot trial to be performed on children less than 2 years of age. Awaiting trial results	[36]

## Data Availability

The data underlying this article will be shared on request to the corresponding author.

## Supplementary Materials

The following supporting information can be downloaded at: https://www.mdpi.com/article/10.3390/jcdd10040148/s1. Search strategy can be downloaded in the Supplementary Material.
