# Supplementary material for: Growing Heart Valve Implants for Children
Source: J Cardiovasc Dev Dis. 2023 Mar 31;10(4):148. doi: 10.3390/jcdd10040148 (PMC10143004; doi:10.3390/jcdd10040148)
Supplement: Supplementary file 1 [file jcdd-10-00148-s001.zip › jcdd-2283323-supplementary.pdf]

SEARCH STRATEGY

PubMed (U.S. National Library of Medicine, National Institutes of Health):
(("Tissue Engineering"[Mesh]) AND "Heart Valve Prosthesis"[Mesh]) AND ("Heart Valves/abnormalities"[Mesh] OR "Heart Valves/growth and development"[Mesh] OR "Heart Valves/surgery"[Mesh] OR "Heart Valves/transplantation"[Mesh])

- Filters/limits: English
- Date searched: October 24, 2022
- Number of records identified: 98

Inclusion criteria: heart valve implants with growth potential, preclinical large animal studies, clinical trials in pediatric patients

Exclusion criteria: mechanical valves, cryopreserved homografts, clinical trials in adult patients
